# Novel Transforming Growth Factor-Beta Receptor 1 Antagonists through a Pharmacophore-Based Virtual Screening Approach

**DOI:** 10.3390/molecules23112824

**Published:** 2018-10-31

**Authors:** Junhao Jiang, Hui Zhou, Qihua Jiang, Lili Sun, Ping Deng

**Affiliations:** College of Pharmacy, Chongqing Medical University, No. 1 Yixueyuan Rd., Yuzhong District, Chongqing 400016, China; 100876@cqmu.edu.cn (J.J.); hzhou@cqmu.edu.cn (H.Z.); 100877@cqmu.edu.cn (Q.J.); 100901@cqmu.edu.cn (L.S.)

**Keywords:** TGF-beta, pharmacophore, antagonists, Discovery Studio, TGFBR, TGFβ

## Abstract

As new drugs for the treatment of malignant tumors, transforming growth factor-beta receptor 1 (TGFβR1) antagonists have attracted wide attention. Based on the crystal structure of TGFβR1-BMS22 complex, the pharmacophore model A02 with two hydrogen bond acceptors (HBAs) and four hydrophobic (HYD) properties was constructed. From the common features of active ligands reported in the literature, pharmacophore model B10 was also generated, which has two aromatic ring centers (RAs) and two HYD properties. The two models have high sensitivity and specificity to the training set, and they are highly consistent in spatial structure. Combining the two pharmacophore models, two novel skeleton structures with potential activity were selected by virtual screening from the DruglikeDiverse, MiniMaybridge, and ZINC Drug-Like databases. Four compounds (YXY01–YXY04) with potential anti-TGFβR1 activity were designed based on the new skeleton structures. In combination with Lipinski’s rules; absorption, distribution, metabolism, excretion, and toxicity (ADMET); and, toxicological properties predicted in the study, YXY01-03 with the novel skeleton, good drug-like properties, and potential activity were finally discovered and may have higher safety relative to BMS22, which may be valuable for further research.

## 1. Introduction

**Figure 1:**
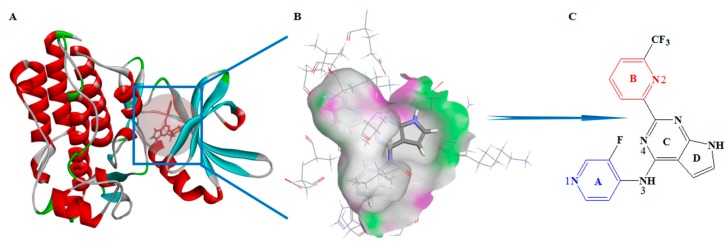
(**A**) Crystal structure of complex (Protein Data Bank (PDB) ID: 6B8Y), (**B**) receptor surface of H-bond, and (**C**) structure of BMS22, of which four rings are labeled A, B, C and D, and four key nitrogen atoms are specified as 1, 2, 3, and 4.

Transforming growth factor-beta (TGFβ) plays a crucial role in regulating cell proliferation, differentiation, and migration in all tissues of the human body [1]. Mature TGFβ is a homodimer consisting of two polypeptide chains that are connected by a disulphide bond and forming a complex with a total molecular weight of 25 kDa. TGFβ induces regulation effects by activating specific receptors on the surface of the cell membrane. Currently, specific receptors for TGFβ include three types, TGFβR1, TGFβR2, and TGFβR3. After the binding of TGFβ to two TGFβR2 subunits, the ligand-receptor complex is recognized by two TGFβR1 subunits and undergoes phosphorylation to form a heterotetrameric receptor complex. The heterotetramer is able to transmit the signal further into the cell and it then activates the Smad signaling pathway [2]. The TGFβ signaling pathway inhibits tumor growth in early-stage tumors. However, in advanced-stage tumors, the high activity state of the pathway changes the cytoskeleton, remodels the matrix, adheres the cell, and migrates gene expression, leading to metastasis of tumor cells [3]. At the same time, the TGFβ signaling pathway mediates primary changes of the tumor microenvironment and induces an epithelial-to-mesenchymal transition, which contributes to cell migration and invasion. The recognition of TGFβR1 by the TGFβ–TGFβR2 complex is the key node of the TGFβ signaling pathway. TGFβR1 signaling is initiated when TGFβ binds to TGFβR2, with the subsequent phosphorylation of Smad2/Smad3 proteins. Blocking its phosphorylation to Smad2/Smad3 can effectively inhibit TGFβ signal transduction to the nucleus [4]. Therefore, TGFβR1 antagonists as new drugs for the treatment of myelodysplastic syndrome, primary hepatocellular carcinoma, glioma, metastatic pancreatic cancer, and other malignant tumors have attracted much attention [5,6]. International pharmaceutical companies, such as GlaxoSmithKline (GSK), Eli Lilly and Company (LLY), and Bristol-Myers Squibb (BMS), have been developing TGFβR1 antagonists in recent years. Among them, a small molecule antagonist of TGFβR1, galunisertib, has entered phase III clinical study, developed by LLY as a new drug mainly for myelodysplastic syndrome, primary hepatocellular carcinoma, glioma, and metastatic pancreatic cancer [7]. In addition, the new TGFβRI antagonists developed by BMS have good clinical application prospects and economic value. BMS has submitted two patent applications for invention in China, numbers 201680055202.3 and 201680049890.2, which have entered substantive examination [8,9]. BMS22 (Figure 1C), one compound of the two patents, has very good selectivity and high activity (half maximal inhibitory concentration (IC_50_) is about 0.55 nM) [10].

Traditional drug research and development techniques are time-consuming and resource-demanding processes. With the development of computer technology, it is very useful to discover and optimize lead compounds by molecular simulation technology to reduce the cycle and cost of drug research and development. Pharmacophore is an abstract description of molecular characteristics, an ensemble of steric and electronic features, which plays a crucial role between the active molecule and the receptor. Based on these features, many diverse chemical compounds can then be virtually screened to find the potent drugs in a fast and convenient way that was impossible for experiments until now. Thus, the pharmacophore-based virtual screening approach has become a powerful and efficient tool in drug research and development [11]. Establishing the pharmacophore model generally includes two methods. One is ligand-based pharmacophore modeling: Based on a series of active ligand structures, conformational analysis and molecular superimposition are performed to obtain an abstract representation that includes key pharmacophore elements and is critical to the active ligands. The other is structure-based pharmacophore modeling, by which a pharmacophore model is constructed according to the steric and chemical features of receptor active sites. If the receptor-ligand complex is obtained, the crystal structure can produce a very accurate pharmacophore model. The pharmacophore modeling strategy has been successfully applied to drug development, such as virtual screening and structural modification [12,13,14].

In this study, pharmacophore models of TGFβR1 antagonists were constructed by the two above-mentioned methods in an attempt to explore a more rapid, comprehensive, and accurate virtual screening approach. Novel skeleton structures with potential activity were selected through pharmacophore-based virtual screening. Following the modification of skeleton structures, novel TGFβR1 antagonists with potentially high activity were designed. This study can provide theoretical guidance for the design of subsequent active anti-TGFβR1 compounds.

## 2. Materials and Methods

### 2.1. Structure-Based Pharmacophore Model Construction

With the development of X-ray diffraction in crystals, cryogenic scanning electron microscopy (Cryo-SEM), and nuclear magnetic resonance (NMR), functional genomics and structural biology have developed rapidly. More and more structures of target receptors and ligand-receptor complexes have been elucidated. Pharmacophore model construction based on the structure of ligand-receptor complex has become an active field.

The crystal structure of TGFβR1 protein was first reported in 1999 (Protein Data Bank (PDB) ID:1B6C) [15]. The core region of TGFβR1 catalytic domain adopts a canonical protein kinase fold, and it consists of a C-terminal with an α-helix and an N-terminal with a β-fold. The deep groove between them is involved an adenosine triphosphate (ATP) binding site to substrate (Figure 1A). Up to now, the PDB database has been loaded with a number of small molecular antagonists and the crystal structures of receptor-ligand complexes (http://www.rcsb.org database), including 1PY5, 1VJY, 2WOT, 2X7O, 3FAA, 3GXL, 3KCF, 3TZM, 6B8Y, and so on. BMS22, which was developed by BMS, is a greatly potent TGFβR1 antagonist with high selectivity and activity [16]. The crystal structure of ligand-receptor complex (PDB ID: 6B8Y) well explains the interaction site and mode between the antagonist and the receptor. The steric and chemical structures of the receptor provide good information for the construction of a pharmacophore model.

The crystal structure of ligand-receptor complex retrieved from the PDB database was used to construct the structure-based pharmacophore model after supplementing amino acid residues, adding hydrogen atoms, and performing other protein preparation processes. The complex crystal structure, receptor surface of H-bond surface, and BMS22 molecular structure are shown in Figure 1. The Receptor–Ligand Pharmacophore Generation module [17] of BIOVIA Discovery Studio 2017R2 (DS 2017R2) was used to generate a three-dimensional (3D) pharmacophore model with default parameters. According to the model scoring value, by combining the sensitivity and specificity of the model to the test set molecule with the receiver operating characteristic (ROC) curve, the best pharmacophore model will be selected.

### 2.2. Ligand-Based Pharmacophore Model Construction

The characteristic structures of a series of active compounds with similar activities but different structures can generate ligand-based pharmacophore models by comparison and superposition. Thus, the best pharmacophore model can be used for virtual screening of a small compounds database to search for potential drugs.

**Figure 2:**
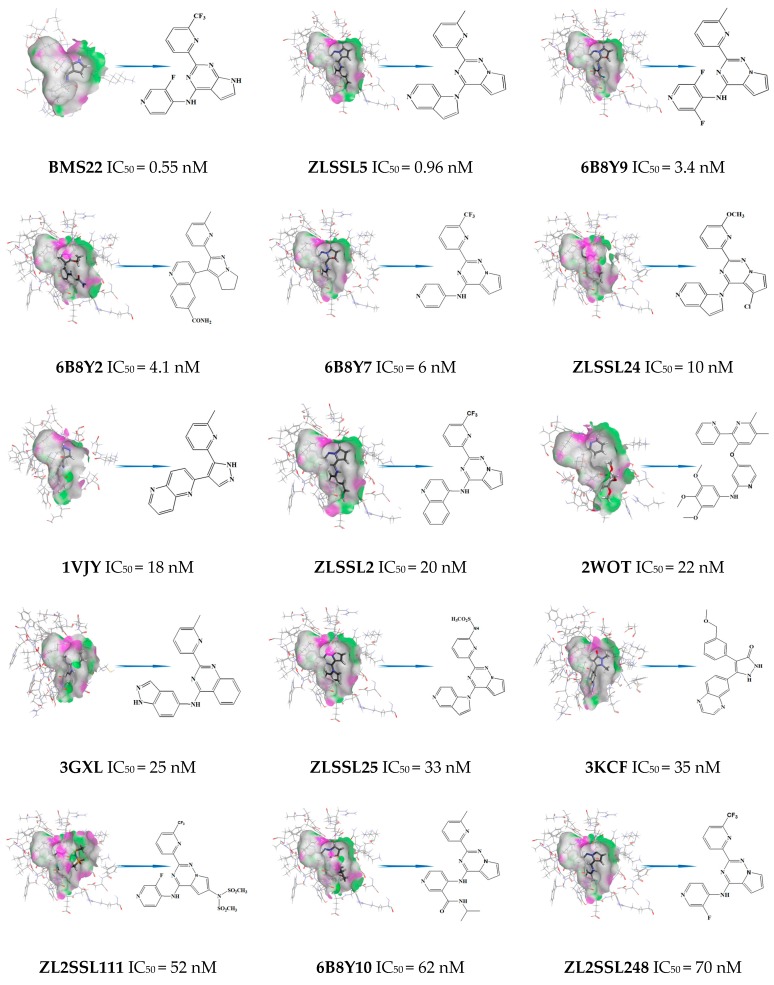

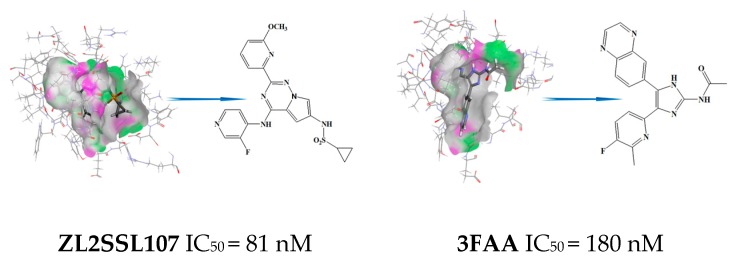
A set of 17 compounds made up the training set used in common feature pharmacophore generation.

Based on the principle of the diversity of active molecular structures of a training set, 17 TGFβR1 antagonists with high activity reported in the literature were selected to construct the training set. The activity against TGFβR1 ranges from 0.55 nM to 180 nM (IC_50_). Six conformations of these compounds, BMS22, 1VJY, 2WOT, 3GXL, 3KCF, and 3FAA, were extracted directly from the crystal structures of the ligand-protein complexes of the PDB database. The other active conformations of the training set cannot be downloaded from the PDB database. Therefore, molecular docking of the 11 compounds was performed by using the DS docking module. According to the docking results, the most active conformations were selected for the construction of the pharmacophore. The molecular structures of the training set are shown in Figure 2. The series of molecules that were named at the beginning of 6B8Y came from the study of Harikrishnan et al. [10]; the series of molecules named at the beginning ZLSSL and ZL2SSL came from patent documents 201680049890.2 and 201680055202.3, respectively; the rest of the molecules came from the PDB database and were named after the database ID. In light of the IC_50_ values of active molecules, we defined the Principal and MaxOmitFeat properties. For molecules with IC_50_ ≤ 10 nM, Principal is defined as 2 for active, in which a reference molecule ensures that all of the chemical features in the molecule are considered in building the pharmacophore space, and Max OmitFeat is defined as 0, i.e., all of the characteristic elements in the pharmacophore model need to be matched with the compounds. For molecules with IC_50_ between 10 and 100 nM, Principal is defined as 1 for moderately active, in which conformations of this molecule are considered, and Max OmitFeat is defined as 1, i.e., one characteristic element in the pharmacophore model can be omitted from the compound.

In the study, the hydrophobic (HYD) characteristics, hydrogen bond acceptor (HBA), hydrogen bond donor (HBD), and aromatic ring center (RA) were selected as the characteristic pharmacophore elements. Using the Common Feature Pharmacophore Generation module of DS 2017R2, the models were constructed based on the active conformations of the training set molecules. When considering the coincidence degree between training set molecule and model combined with ROC curve, a suitable pharmacophore model was selected.

### 2.3. Evaluation and Validation of Pharmacophore Models

Pharmacophore model validation was performed to evaluate the models’ reliability and quality. In this paper, three paths were used to evaluate and verify the pharmacophore models. (1) The models were validated using the group of test set compounds. Accurate test data prediction is an important attribute of pharmacophore model reliability. The group of test set compounds should include both active and inactive molecules, so it can select the best pharmacophore model with high distinguishing ability between known active and inactive compounds. In total, 87 compounds showing experimental anti-TGFβR1 activity were selected from the literature to validate the sensitivity (SE) of models. SE is defined as the ratio of true positive (TP) to the sum of TP and false negative (FN): [TP/(TP + FN)]. Inactive molecules were downloaded from the DUD-E database (http://dude.docking.org/), which includes 102 drug action targets [18]. The specificity (SP) of the TGFβR1 was verified by using 8677 inactive molecular models that are provided in the DUD-E database. SP is defined as the ratio of true negative (TN) to the sum of TN and false positive (FP): [TN/(TN + FP)]. SE and SP values represent the ability of the model to recognize both active and inactive molecules. The closer the value is to 1, the stronger the recognition ability. (2) The pharmacophore models would have higher reliability, which was more consistent with the characteristics of structure-activity relationships obtained from the experiments. (3) The key amino acids in the active sites of TGFβR1 were obtained. The chemical characteristics that were necessary for both the pharmacophore model and these key amino acids were comprehensively analyzed.

### 2.4. Virtual Screening of Databases

Pharmacophore models were used as 3D queries to screen the compounds from two database sources. One source was the DruglikeDiverse and MiniMaybridge databases, including 5384 and 2000 compounds, respectively. The other was the ZINC Drug-Like database, which contains 147,808 compounds based on druglike data.

According to the results of virtual screening and the experimental structure-activity relationship, combined with the reality and rationality of synthesis, novel TGFβR1 antagonists were designed. Finally, absorption, distribution, metabolism, excretion, and toxicity (ADMET) parameters were computed for all screened compounds with maximum fit values and minimum IC_50_ values. We identified the novel structure of TGFβR1 antagonists while considering the results of ADMET as well as Lipinski’s rules. This study lays a foundation for the following chemical synthesis and evaluation of pharmacodynamics.

## 3. Results and Discussion 

### 3.1. Characteristics and Reliability Verification of Ligand–Receptor Complex Pharmacophore Model

**Figure 3:**
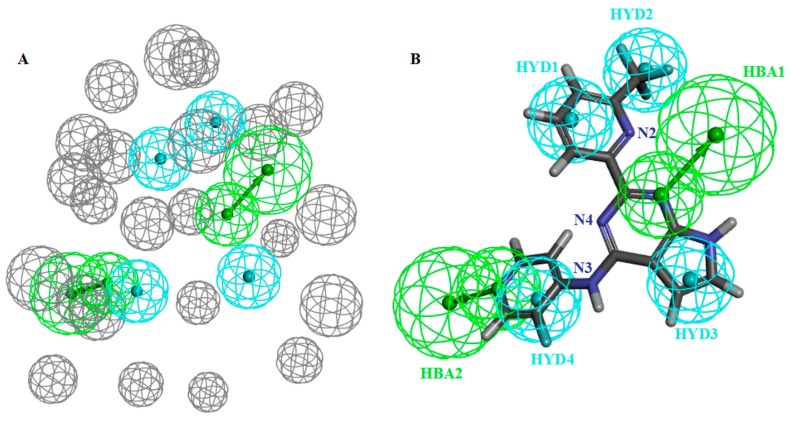
(**A**) Pharmacophore model A02 (hydrogen bond acceptors in green balls; hydrophobic properties in blue balls; the exclude volumes in gray balls) and (**B**) superposition with compound BMS22 (gray balls were hidden).

Ten pharmacophore models were constructed, and the related data are shown in Table 1. Combining selectivity scoring value, SE value, and SP value with the area under the ROC curve, model A02 was selected as the finest model (shown in Figure 3A). The pharmacophore model was characterized by AAHHHH, containing two HBAs and four HYDs (Figure 3B). Model A02 had high sensitivity (SE = 0.81176) and high specificity (SP = 0.78754) to the group compounds of the training set. The area under the ROC curve was characterized by quality value, which was 0.879. The structure-activity relationship obtained from the experimental study confirmed that the presence of -CF_3_ on the pyridine ring B (Figure 1, Ring B) of BMS22 would significantly enhance the selectivity of the compound to TGFβR1, which could be an important pharmacodynamic feature, but the addition of polar groups (such as carbonyl group) to pyridine ring A (Figure 1, Ring A) would significantly decrease the activity of BMS22. Thus, the hydrophobicity of ring A of BMS22 is also an important pharmacodynamic characteristic. Two hydrogen bond characteristics existed in the crystal complex, which showed a crucial interaction of the active site. In general, model A02 well reflected the important characteristics that were found in the study of structure-activity relationship, and it was more reliable.

Based on above analysis, we selected model A02 as the final ligand-receptor complex pharmacophore model for further study.

In the crystal structure of ligand-receptor complex, N3 and N4 atoms of BMS22 are far away from the receptor and do not form an intermolecular hydrogen bond. In the case of drug design, structural modification can be considered at this site.

### 3.2. Characteristics and Reliability Verification of Ligand-Based Pharmacophore Model

Ten ligand-based pharmacophore models were also constructed, and the related parameters are shown in Table 2. When combining rank value, SE value, and SP value with the area under the ROC curve, model B10 was selected as the final model.

**Figure 4:**
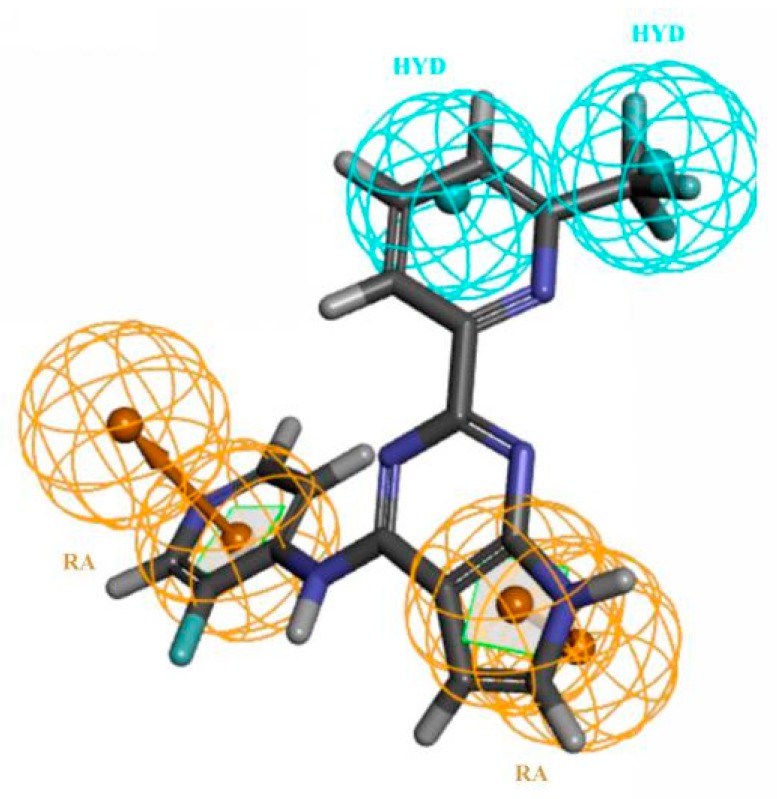
Pharmacophore model B10 superposed with BMS22 (aromatic ring centers in orange balls; hydrogen bond acceptors in blue balls).

Model B10 was characterized by RRYY, containing two RAs and two HYDs (shown in Figure 4). Model B10 had high sensitivity (SE = 0.92941) and high specificity (SP = 0.73299) to the group compounds of the training set, and the quality value of the model was 0.950. The pharmacophore model and the training set molecule matched well, so the reliability of the model was high.

Thus, model B10 was selected as the final ligand-based pharmacophore model for further study.

**Figure 5:**
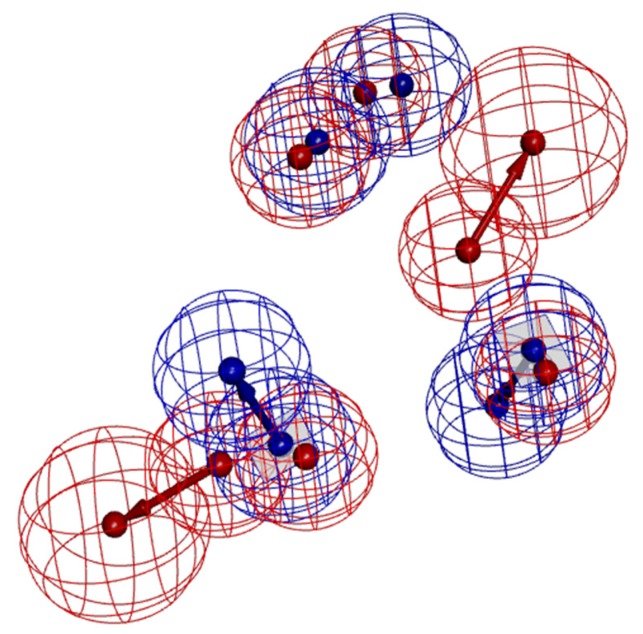
Comparison of model A02 (red) and B10 (blue) pharmacophores.

When compared to model A02, model B10 had fewer pharmacodynamic characteristics and was generally more sensitive to active molecules. In order to compare the differences between them, model A02 and model B10 were superimposed, as shown in Figure 5. According to the spatial distribution of the pharmacodynamic characteristics of the two models, two hydrophobic characteristics of model B10 overlapped with two hydrophobic characteristics of model A02, and two aromatic rings of model B10 coincided with the other two hydrophobic characteristics of model A02. It can be seen that the two models had high similarity. Model A02 was inconsistent, with two hydrogen bond receptor characteristics. From the point of view of pharmacodynamic activity, the three most active molecules, BMS22, ZLSSL5, and 6B8Y9, all had two hydrogen bond receptor characteristics. Therefore, in the virtual screening, model B10 was used for primary screening, and then model A02 was used for fine screening to screen antagonists with higher activity.

### 3.3. Virtual Screening and Structural Transformation of Lead Compounds

**Figure 6:**
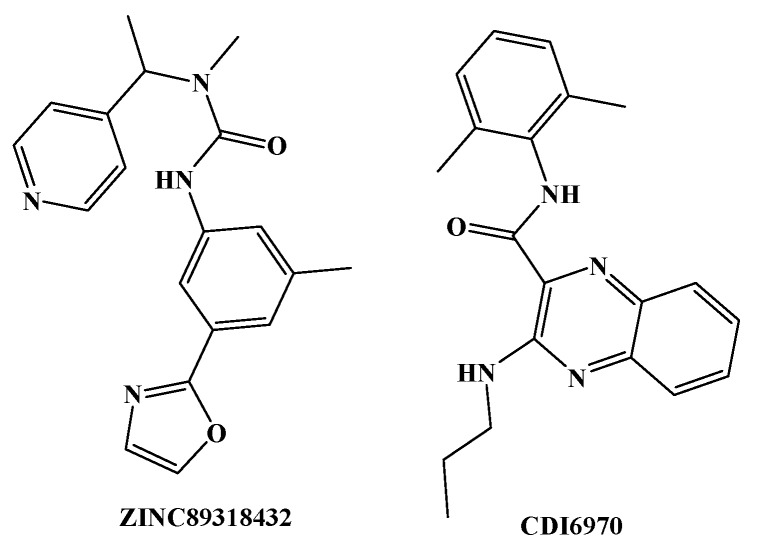
Two compounds with great structural difference from BMS22 were obtained based on Fitvalue value (ZINC89318432 comes from ZINC Drug-Like database and CDI6970 comes from DruglikeDiverse database).

Model B10 was used to screen the DruglikeDiverse, MiniMaybridge, and ZINC Drug-Like databases, and 950, 350, and 8824 compounds were obtained, respectively. Based on the virtual screening results, the above compounds were matched with model A02 by using the Ligand Profiler module. Two compounds with great structural difference from BMS22 were obtained according to the Fitvalue value, and the structures of which are shown in Figure 6. On the basis of the structural characteristics of the selected compounds and their matching with the pharmacophore model, a series of compounds were designed combining the experimental results of structure-activity relationship.

**Figure 7:**
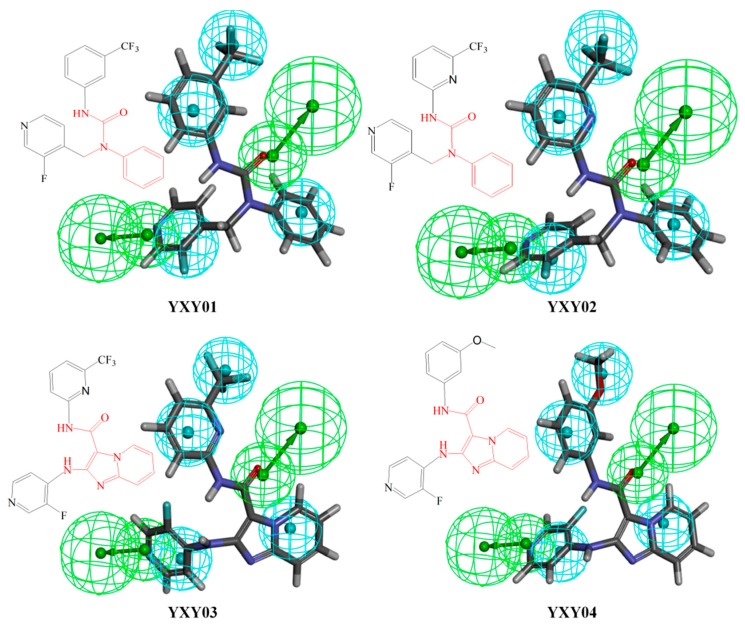
Compounds YXY01–04 obtained by virtual screening using pharmacophore model A02 (hydrogen bond acceptors in green balls; hydrophobic properties in blue balls).

The self-designed series of compounds were matched with model A02 again. Finally, four potentially active compounds (YXY01–YXY04) were obtained according to the Fitvalue. These molecules are depicted in Figure 7. YXY01 and YXY02 have the same skeleton structure, and YXY03 and YXY04 have the same skeleton structure. All four compounds have distinct molecular skeleton structures as compared with the related patents of BMS, which can break through the relevant patent protection.

The four compounds were evaluated by Lipinski’s rule, and all were in accordance with the relevant rules. Lipinski’s rule states that compounds are likely to have good absorption and permeation in biological systems and are thus likely to be successful drug candidates if they meet the following criteria: (1) molecular weight (MW) is less than 500 Dalton; (2) the number of hydrogen bond acceptors (HBAs) is fewer than 10; (3) the number of hydrogen bond donors (HBDs) is fewer than 5; (4) the number of rotatable bonds (ROTBs) does not exceed 10; and, (5) calculated Log*P* (oil-water partition coefficient) is less than 5. The properties of ADMET of the four designed compounds and BMS22 were predicted using DS 2017R2. The results show that the water solubility of the four compounds was in the order of YXY01 ≈ YXY02 ≈ YXY04 > BMS22 ≈ YXY03. YXY03, YXY04, and BMS22 had moderate blood-brain barrier transmittance, while YXY01 and YXY02 had higher blood-brain barrier permeability; neither YXY01–04 nor BMS22 had cytochrome P450 2D6 inhibition; and, both YXY01–04 and BMS22 had very good intestinal absorption. 

The parameters of Lipinski’s rule and the predicted results of important toxicological properties are listed in Table 3. The data indicate that none of the compounds had mutagenicity except YXY04; YXY01–04 and BMS22 had no potential developmental toxicity; YXY01–04 and BMS22 had no potential carcinogenicity in female mice, but all of the compounds had carcinogenicity in male mice, which needs further evaluation in a future study; the calculation of lowest observed adverse effect level (LOAEL) showed that YXY03 and YXY04 had higher doses than BMS22; YXY03 had similar maximum tolerated doses (MTDs) to BMS22, while other candidates had a little lower MTD value than BMS22; the prediction results reveal drug median lethal dose (LD_50_) of YXY01–04 are one to two orders of magnitude higher than that of BMS22, which shows that the designed compounds may have higher safety.

In conclusion, the structures of the designed compounds were novel and the skeleton structures were significantly different from those of TGFβR1 antagonists that are reported at present. The predicted results of Lipinski’s rule, ADMET, and toxicological properties indicate that YXY01–03 are worthy of further study because of their potentially higher safety than BMS22.

## 4. Conclusions

In this study, reliable pharmacophore models A02 and B10 were constructed by two modeling methods that are based on the crystal structure of BMS22-TGFβR1 complex and a group of compounds with anti-TGFβR1 activity reported in the literature, respectively. The latter was used for primary screening and the former for fine screening. The combination of the two pharmacophore construction methods is conducive to rapid, comprehensive, and accurate screening of highly active candidate compounds. Two new skeleton structures were found by searching the databases, and subsequently three compounds (YXY01–03) with certain activity and high safety were designed. The activity of the compounds could be further predicted by molecular docking, and the potentially active compounds could be synthesized and evaluated.

## Figures and Tables

**Table 1:** Features, selectivity scores, and validation results of the receptor-ligand-based pharmacophore models. TP, true positive; TN, true negative; FP, false positive; FN, false negative; SE, sensitivity; SP, specificity.

Model	Features	Selectivity Score	Total Actives	Total Inactives	TP	TN	FP	FN	SE	SP
A01	AADHHH	10.516	85	8397	13	7820	577	72	0.15294	0.93128
A02	AAHHHH	9.6023	85	8397	69	6613	1784	16	0.81176	0.78754
A03	AADHH	9.0011	85	8397	30	6542	1855	55	0.35294	0.77909
A04	ADHHH	9.0011	85	8397	60	5688	2709	25	0.70588	0.67738
A05	AADHH	9.0011	85	8397	18	6984	1413	67	0.21176	0.83173
A06	ADHHH	9.0011	85	8397	20	7275	1122	65	0.23529	0.86638
A07	AADHH	9.0011	85	8397	24	7059	1338	61	0.28235	0.84066
A08	AAHHH	8.0875	85	8397	72	3865	4532	13	0.84706	0.46028
A09	AAHHH	8.0875	85	8397	80	4677	3720	5	0.94118	0.55698
A10	AAHHH	8.0875	85	8397	69	3497	4900	16	0.81176	0.41646

**Table 2:** Feature set, selectivity score, and validation results of the ligand-based pharmacophore models.

Model	Features	Rank	Total Actives	Total Inactives	TP	TN	FP	FN	SE	SP
B01	ARHH	135.897	85	8397	81	2315	6082	4	0.95294	0.27569
B02	AHHH	134.118	85	8397	83	4554	3843	2	0.97647	0.54243
B03	RRHH	133.760	85	8397	79	6427	1970	6	0.92941	0.76539
B04	RRHH	133.760	85	8397	79	6456	1941	6	0.92941	0.76885
B05	ARHH	132.197	85	8397	80	2325	6072	5	0.94118	0.27688
B06	RHHH	132.169	85	8397	79	6121	2276	6	0.92941	0.72895
B07	RHHH	130.718	85	8397	82	4558	3839	3	0.96471	0.54281
B08	RHHH	129.478	85	8397	79	5872	2525	6	0.92941	0.69930
B09	RRHH	129.283	85	8397	79	6370	2027	6	0.92941	0.75860
B10	RRHH	128.545	85	8397	79	6149	2248	6	0.92941	0.73229

**Table 3:** Parameters of Lipinski’s rule and toxicity prediction of compounds YXY01–04 and BMS22 calculated by DS TOPKAT. MW, molecular weight; ROTB, rotatable bond; HBA, hydrogen bond acceptor; HBD, hydrogen bond donor; DTP, developmental toxicity potential; LOAEL, lowest observed adverse effect level; MTD, maximum tolerated dose; LD_50_, median lethal dose.

	MW	Log*P*	ROTB	HBA	HBD	Mutagenicity	DTP	Carcinogenicity (Female)	LOAEL (g/kg)	MTD (Feed, g/kg)	LD_50_ (Oral, g/kg)
BMS22	374	3.956	4	5	2	Nonmutagen	Nontoxic	Noncarcinogen	0.0064	0.137	0.0413
YXY01	389	4.309	5	2	1	Nonmutagen	Nontoxic	Noncarcinogen	0.0037	0.069	0.863
YXY02	390	4.126	5	3	1	Nonmutagen	Nontoxic	Noncarcinogen	0.0027	0.075	0.274
YXY03	416	3.687	5	5	2	Nonmutagen	Nontoxic	Noncarcinogen	0.0101	0.143	0.42
YXY04	377	2.911	5	5	2	Mutagen	Nontoxic	Noncarcinogen	0.0185	0.104	1.02
